# Time trends in leisure time physical activity and physical fitness in elderly people: 20 year follow-up of the Spanish population national health survey (1987-2006)

**DOI:** 10.1186/1471-2458-11-799

**Published:** 2011-10-13

**Authors:** Domingo Palacios-Ceña, Cristina Alonso-Blanco, Rodrigo Jiménez-Garcia, Valentin Hernández-Barrera, Pilar Carrasco-Garrido, Elena Pileño-Martinez, Cesar Fernández-de-las-Peñas

**Affiliations:** 1 Department of Health Science II, Universidad Rey Juan Carlos, Madrid, Spain; 2 Preventive Medicine and Public Health Teaching and Research Unit, Department of Health Sciences, Universidad Rey Juan Carlos, Madrid, Spain; 3 School of Public Health. Madrid. Spain; 4 Department of Physical Therapy, Occupational Therapy, Rehabilitation and Physical Medicine, Universidad Rey Juan Carlos, Alcorcón, Madrid, Spain

## Abstract

**Background:**

To estimate trends in leisure time physical activity and physical fitness between 1987-2006 in older Spanish people.

**Methods:**

We analyzed data collected from the Spanish National Health Surveys conducted in 1987 (n = 29,647), 1993 (n = 20,707), 1995-1997 (n = 12,800), 2001 (n = 21,058), 2003 (n = 21,650), and 2006 (n = 29,478). The number of subjects aged ≥ 65 years included in the current study was 29,263 (1987: n = 4,958-16.7%; 1993: n = 3,751-17.8%; 1995-97: n = 2,229-17.4%; 2001: n = 4,356-20.7%; 2003: 6,134-28.3%; 2006: 7,835-26.5%). Main variables included leisure-time physical activity and physical fitness. We analyzed socio-demographic characteristics, self-rated health status, lifestyle habit and co-morbid conditions using multivariate logistic regression models.

**Results:**

Women exhibited lower prevalence of leisure time physical activity and physical fitness compared to men (P < 0.05). The multivariate analysis for time trends found that practising leisure time physical activity increased from 1987 to 2006 (P < 0.001). Variables associated with a lower likelihood of practicing leisure time physical activity were: age ≥ 80 years old, ≥ 2 co-morbid chronic conditions, and obesity. Variables associated with lower physical fitness included: age ≥ 80 years, worse self rated health; ≥ 2 medications (only for walking), and obesity.

**Conclusions:**

We found an increase in leisure time physical activity in the older Spanish population. Older age, married status, co-morbid conditions, obesity, and worse self-perceived health status were associated with lower activity. Identification of these factors can help to identify individuals at risk for physical inactivity.

## Background

In recent years, there has been an increase of aging in the society [1]. The aging of the population can lead to an increase in the number of individuals at risk for chronic diseases [2]. In an article from the Center for Disease Control and Prevention's Healthy Aging Network, physical activity (PA) was considered one key element for determining health status [3]. Recent guidelines include PA recommendations for older people [4] because regular PA can provide health benefits, even when it is initiated later in life [5]. In fact, evidence suggests that PA is associated with more years of life, self-perceived healthy life, years without impairment in daily live activities [6], lower rates of functional decline [7], lower risk of mortality [8,9], increased longevity [6,10], reduced risk of type 2 diabetes [11], and better quality of life [12].

Physical activity is defined as any bodily movement produced by skeletal muscles that result in energy expenditure [13]. Nevertheless, physical activity is a broad term that encompasses both leisure-time activity (sports, exercise) [13] and activities of daily life [13,14]. Leisure time physical activity (LTPA) refers to conditioning exercise or sports not related to regular work activities [13,15]. Walking is the most common form of physical activity and is recommended for all ages [16-18]. Physical fitness is defined as a set of attributes that people have or achieve that relates to the ability to perform physical activity [13]. In fact, different studies have used walking and walking up-stairs to evaluate physical fitness of older people [19,20]. However, PA research has mainly focused on middle-aged and the elderly combined [17,21-28]. In fact, few studies have investigated PA only in older people [14,16,29-33].

Studies conducted in the USA [28], Australia [21], England [27] and Scotland [22] had reported a trend towards an increased PA in individuals older than 60 years of age. In fact, the increase in PA has been found to be higher in people older than 65 years than in middle-aged population [17,25,33]. Nevertheless, some authors have suggested the opposite, that older people report lower PA [21,23,26]. In line with this hypothesis, the Center for Disease Control [24] reported that the prevalence of LTPA declined from 29.8% in 1994 to 23.7% in 2007 in the United States.

In Spain, more than 40% of older adults are sedentary [34-37]. Although the percentage of people who practice LTPA has increased [36], more information is needed to understand factors that facilitate or inhibit older people tendency to engage in LTPA. Previous studies conducted in older adults have reported that important variables for PA include those potentially handled from public health and social-educational policies: gender [16,29,33], age [16,22,29], educational level [14,32,33], monetary income [14,17,32], marital status [29], co-morbid diseases [16,17,29], alcohol consumption [16,17], smoking [17,29], self-perceive health [30,33], and obesity [14,22,31].

No previous study has examined the time trends of physical activity in the last 20 years in older Spanish people. Therefore, the current study examines time trends in prevalence of PA for adults aged 65 and over using Spanish National Health Surveys (SNHS) conducted in the period 1987-2006. The objectives of this study were: 1) to describe the prevalence of LTPA and physical fitness among the Spanish elderly population in the period 1987-2006; 2) to determine socio-demographic features, self-perceived health status, co-morbidity, and lifestyle-related habits associated with LTPA and physical fitness in older people; and, 3) to analyze time trends in prevalence of LTPA and physical fitness in the period 1987-2006 in Spanish older people.

## Methods

### Ethical aspects

As this analysis was conducted on a de-identified, public-use dataset it was not necessary to have the approval of an ethics committee according to Spanish legislation.

### The Spanish National Health Surveys (SNHS)

We conducted a cross-sectional study using individualized data obtained from the SNHS done in 1987, 1993, 1995, 1997, 2001, 2003, and 2006. The SNHS is an ongoing, home-based personal interview examining a national representative sample of non-institutionalized population residing in main family dwellings (households) of Spain and is mainly performed by the Ministry of Health and Consumer Affairs and the National Statistics Institute (Instituto Nacional Estadística-INE). The SNHS uses a multistage cluster sampling, with proportional random selection of primary and secondary sampling units (towns and sections, respectively), with the final units (individuals) being selected by means of random routes and sex- and age-based quotas.

Surveyors were previously trained about basic communication skills, procedures and the used questionnaire. Informed consent was signed by all participants before they answered the survey. In order to meet the surveys' stated aim of being able to furnish estimates with a certain degree of reliability at both national and regional levels the following samples of adult aged 15 years and older were selected in the SNHS: 29,647 in 1987; 20,707 in 1993; 21,058 in 2001; 21,650 in 2003; and 29,478 in 2006. Surveys conducted in 1995 and 1997 were based on smaller sample sizes (N = 6,400), therefore these two databases were joined and analyzed together. The number of subjects aged ≥ 65 years included in the study along the entire period was 29,263 (1987: n = 4,958-16.7%; 1993: n = 3,751-17.8%; 1995-97: n = 2,229-17.4%; 2001: n = 4,356-20.7%; 2003: 6,134-28.3%; 2006: 7,835-26.5%). More details about the SNHS methodology are described elsewhere [38,39].

For the purpose of the current study, we included answers from adults aged 65 years and older from these 7 SNHS. The variables included in the current study were created on the basis of several questions included in the questionnaires and identical in all surveys. The dependent variables were: 1, LTPA, which was collected using the following question: "Do you practice any physical activity during your leisure time?", with 2 possible answers: "none" or "once a month or more", and 2, physical daily fitness, which was assessed with 2 questions: "Can you walk up 10 steps without help?" and, "Can you keep walking for one hour without rest?." The answer to both questions could be "yes" or "no". These last two questions were first collected within the 1993 survey.

We also analyzed socio-demographic characteristics such as age (65 to 79 years, 80 years and older), marital status (married or living as a couple, unmarried/widow/divorced), and educational level (no study, primary education completed, secondary education, or more).

Self-perceived health status was assessed with the following question: "How did you self-perceive your health status over the previous 12 months?" Subjects described their health status as very good, good, fair, poor, very poor. The answer was dichotomized into very good/good or fair/poor/very poor self-perceived health status. We also collected the number of medical doctor diagnoses of co-morbid chronic conditions (high blood pressure, diabetes, chronic heart disease, chronic bronchitis, emphysema, or asthma) as follows: none, one, two, or more. The number of prescribed medications for any of these chronic conditions was also categorized as none, one, two or more. Body mass index (BMI) was calculated from self-reported body weight and height. Individuals with a BMI ≥ 30 were classified as obese, those with BMI between 25 and 29.9 were classified as overweight and those with BMI < 25 were considered to have normal weight. Individuals with BMI < 18.5 or incomplete data on height and weight were excluded for the analysis.

Regarding lifestyle habits, smoking habits differentiated between current smokers, non-smokers or ex-smokers. Finally, sleep habits were divided into subjects sleeping > 8 hours per day and those sleeping < 8 hours per day.

### Statistical analysis

In this study we analyzed physical activity and physical fitness separately for men and women and we excluded respondents with missing data for any outcome. We calculated descriptive measures for all variables of interest by aged-group and SNHS. Second, we compared the reported prevalence for the dependent variables and age group according to the SNHS. Third, we fit logistic regression models by gender to assess factors independently associated for each dependent variable. Finally, to evaluate the time trend across the period 1987-2006, adjusted odds ratios (ORs) with their confidence intervals were estimated using multivariate logistic regression models. Models were initially adjusted by age and by those variables that yield significant associations within the bivariate analysis. We assessed significant interaction terms in fully adjusted models; for significant effects, we stratified the fully adjusted models by the relevant factor. The estimates were made using the "svy" (survey command) functions of the STATA program, which allowed us to incorporate the study design and weights in all our statistical calculations. Statistical significance was established at P < 0.05 (two-tailed P values).

## Results

The mean age increased significantly from 72.3 to 74.8 years for women and from 72.2 to 74.5 years for men across the study period (P < 0.05). Women were slightly, but significantly older than men in all surveys (P < 0.05). Tables 1, 2 summarize the distribution by socio-demographic characteristics and health related variables among women and men according to the SNHS conducted (1987 2006).

**Table 1:** Frequencies Statistic for WOMEN: Spanish National Health Surveys (SNHS) 1987, 1993, 1995-7, 2001, 2003 and 2006.

		SNHS 1987	SNHS 1993	SNHS 95-97	SNHS 2001	SNHS 2003	SNHS 2006
		
		N = 2,846	N = 2,137	N = 1,303	N = 2,494	N = 3,830	N = 5,022
Age Mean (SE)^+^		72.3 (0.15)	74.3 (0.23)	72.6 (0.17)	73.0 (0.14)	74.7 (0.16)	74.8 (0.14)

Age group^+^	65-79	85.5	84.5	84.9	83.6	76.2	75.5
	
	≥ 80	14.5	15.5	15.1	16.4	23.8	24.5

Marital status*	Unmarried/widow/divorced	54.0	47.9	49.8	47.5	50.9	49.4
	
	Married or living with couple	46.0	52.1	50.2	52.5	49.1	50.6

Educational level*	No studies	72.9	49.7	38.9	26.5	42.0	42.0
	
	Primary education completed	21.7	42.7	55.2	67.2	46.3	45.2
	
	Secondary education or more	5.4	7.6	5.9	6.3	11.7	12.8

Self rated health	Very good/good	34.6	39.4	37.0	36.5	33.0	33.1
	
	Fair/poor/very poor	65.4	60.6	63.0	63.5	67.0	66.9

Nª of chronic conditions*	None	40.8	41.3	37.6	32.9	26.8	19.0
	
	1	31.8	33.0	32.3	33.2	33.2	32.7
	
	≥2	27.4	25.7	30.1	33.9	40.0	48.3

Number of medications*	None	26.7	26.1	19.0	15.4	9.1	6.5
	
	1	34.5	33.6	33.1	31.2	21.4	14.7
	
	≥ 2	38.8	40.3	47.9	53.4	69.5	78.8

BMI*	Normal	41.6	39.1	49.7	31.2	35.2	31.5
	
	Overweight	41.9	43.4	31.4	41.8	42.3	42.4
	
	Obesity	16.5	17.5	18.9	27.0	22.5	26.1

Smoking habits*	Smoker	1.9	3.9	1.7	2.2	1.7	3.1
	
	Ex Smoker	2.7	2.5	2.4	2.9	2.9	4.5
	
	Non Smoker	95.4	93.6	95.9	94.9	95.4	92.4

Sleep habits (hours day)	< 8	45.8	48.2	41.9	42.8	44.8	46.8
	
	≥ 8	54.2	51.8	58.1	57.2	55.2	53.2

Data are expressed as percentages (%)

^+ ^Significant differences between SNHS/*Significant differences between SNHS (adjusted by age)

**Table 2:** Frequencies Statistic for MEN: Spanish National Health Surveys (SNHS) 1987, 1993, 1995-7, 2001, 2003 and 2006

		SNHS 1987	SNHS 1993	SNHS 95-97	SNHS 2001	SNHS 2003	SNHS 2006
		
		N = 2,112	N = 1,614	N = 926	N = 1,862	N = 2,304	N = 2,813
Age Mean (SE)^+^		72.2 (0.18)	74.9 (0.28)	73.3 (0.21)	73.3 (0.16)	73.8 (0.19)	74.5 (0.16)

Age group^+^	65-79	85.3	84.7	84.6	84.4	80.5	77.3
	
	≥ 80	14.7	15.3	15.4	15.6	19.5	22.7

Marital status	Unmarried/widow/divorced	20.8	22.7	20.2	19.4	19.0	20.0
	
	Married or living with couple	79.2	77.3	79.8	80.6	81.0	80.0

Educational level*	No studies	57.9	32.3	34.7	21.2	34.8	30.4
	
	Primary education completed	29.7	49.9	52.6	66.3	44.5	45.5
	
	Secondary education or more	12.4	17.8	12.7	12.5	20.7	24.1

Self rated health*	Very good/good	45.0	48.4	44.5	47.6	44.9	48.5
	
	Fair/poor/very poor	55.0	51.6	55.5	52.4	55.1	51.5

Nª of chronic conditions*	None	44.5	47.4	40.9	35.6	33.0	21.6
	
	1	32.3	34.2	33.9	33.7	33.5	32.3
	
	≥2	23.2	18.4	25.2	30.7	33.5	46.1

Number of medications*	None	37.7	36.4	26.3	22.1	15.0	12.3
	
	1	35.0	37.7	37.0	34.1	30.6	22.8
	
	≥ 2	27.3	25.9	36.7	43.8	54.4	64.9

BMI*	Normal	45.1	37.8	40.3	30.9	27.7	28.0
	
	Overweight	44.5	48.1	45.1	51.8	53.1	51.0
	
	Obesity	10.4	14.1	14.6	17.3	19.2	21.0

Smoking habits*	Smoker	33.8	27.9	24.2	19.3	16.8	15.4
	
	Ex Smoker	43.3	43.9	50.0	53.1	52.2	54.3
	
	Non Smoker	22.9	28.2	25.8	27.6	31.0	30.3

Sleep habits (hours/day)	< 8	59.5	57.3	56.8	52.3	61.0	58.0
	
	≥ 8	40.5	42.7	43.2	47.7	39.0	42.0

Data are expressed as percentages (%)

^+ ^Significant differences between SNHS/*Significant differences between SNHS (adjusted by age)

Among women, the prevalence of those married, higher education, higher number of chronic conditions and medications, obesity and smoking habit significantly increased along the period 1987-2006 (P < 0.01). Among men, the evolution was very similar to women except for smoking habits that decreased from 33.8% to 15.4% (P < 0.01).

Time trends for LTPA, capacity to walk up ten steps without help and to walk for one hour without rest by aged-group and gender are summarized in Table 3. Overall, women exhibited lower prevalence of LTPA and physical fitness (in both variables) as compared to men in all surveys (P < 0.01). In both gender, the prevalence for all dependent variables were always higher in the younger aged group. The highest prevalence of LTPA was found for both genders in the SNHS conducted in 2006, with 54.6% for women, and 69.6% for men, respectively (P < 0.05).

**Table 3:** Time trends by gender and age group in leisure time physical activity and physical fitness between 1987 and 2006

WOMEN		Age group	SNHS 1987	SNHS 1993	SNHS 95-97	SNHS 2001	SNHS 2003	SNHS 2006	P-value*
	
	Leisure time physical activity+	65-79	13.3	28.2	36.8	40.4	36.4	59.2	< 0.001
		
		≥ 80	9.1	23.1	26.0	26.9	18.3	40.3	< 0.001
		
		Total	12.7	24.2	35.1	38.2	32.1	54.6	< 0.001
	Walking up 10 step+	65-79	NA	86.5	87.9	87.6	88.2	85.6	0.056
		
		≥ 80	NA	68.4	70.7	71.2	61.6	66.4	0.108
		
		Total	NA	83.8	85.3	85.0	81.8	80.9	0.401

	Walking for one hour+	65-79	NA	77.8	75.8	79.0	78.5	75.5	0.065
		
		≥ 80	NA	53.6	44.4	53.5	42.6	45.3	0.385
		
		Total	NA	74.2	71.0	75.0	70.0	68.1	0.172

MEN	Leisure time physical activity	65-79	26.6	45.2	56.7	60.5	45.0	73.0	< 0.001
		
		≥ 80	18.9	49.0	41.7	46.8	33.1	58.4	< 0.001
		
		Total	25.5	46.0	53.9	58.4	42.6	69.6	< 0.001
	
	Walking up 10 step	65-79	NA	92.9	93.8	94.5	92.6	91.0	0.158
		
		≥ 80	NA	80.5	82.5	82.9	77.0	74.7	0.502
		
		Total	NA	91.4	91.8	92.7	89.6	87.3	0.056
	
	Walking for one hour	65-79	NA	87.7	88.6	88.3	87.8	84.7	0.072
		
		≥ 80	NA	75.3	66.2	70.6	65.7	61.2	0.248
		
		Total	NA	86.3	84.8	85.7	83.4	79.4	0.066

P value for association between the prevalence of study variables and the SNHS (multivariate regression models)/NA: Not available.

+ Significant differences in the total prevalence of study variables between women and men

Crude time trends analysis by aged-group and gender revealed an increase in the prevalence of LTPA over time among women and men in all aged-groups (P < 0.001). On the contrary, no significant changes for physical fitness during the time period by gender or aged-group were found (P > 0.05).

**Figure 1:**
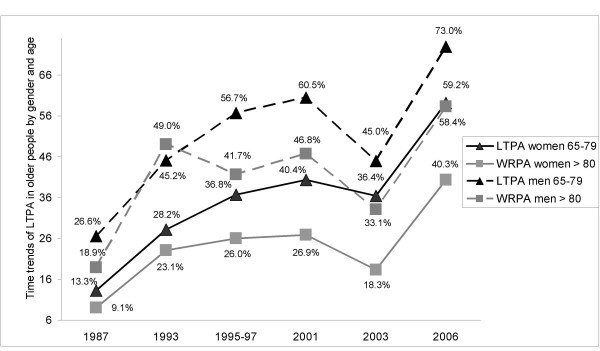
**Time trends of Leisure Time Physical Activity (LTPA)**.

**Figure 2:**
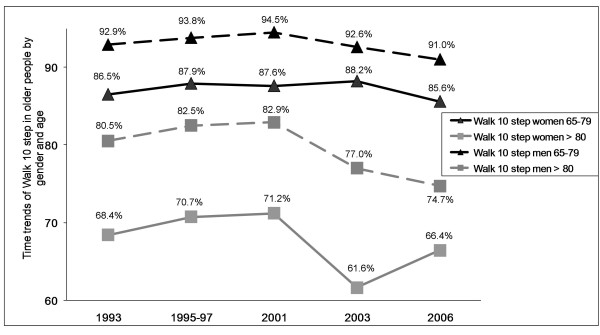
**Time trends of Walk 10 steps**.

**Figure 3:**
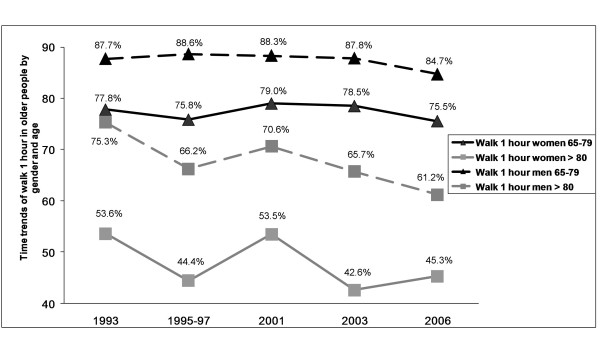
**Time trends of Walk 1 hour**.

The multivariate analysis for time trends in women found that LTPA increased significantly from 1987 to 2006 (P < 0.001, Figure 1). In addition, time trends (1993-2006) for the variable walking for one hour, but not for walking 10 steps without help (Figure 2) also exhibited a significant improvement (P < 0.01, Figure 3). The results of the multivariate analysis to estimate time trends and associated factors for older women are summarized in the table 4. Further, variables significantly associated with a lower likelihood of reporting LTPA among women were: age ≥ 80 years, ≥ 2 co-morbid chronic conditions, and obesity. Variables associated with not being able to walk up ten steps or walking for one hour included: age ≥ 80 years, worse self-rated health, ≥ 2 medications (only for walking for one hour) and again obesity.

**Table 4:** Logistic Regression Models for WOMEN

		Leisure time physical activity	Walking up 10 step	Walking for one hour
Age group	65-79	1	1	1
	
	≥ 80	0.43 (0.36-0.51)	0.27 (0.22-0.34)	0.23 (0.19-0.28)

Marital status	Unmarried/widow/divorced	1	1	1
	
	Married or living with couple	0.87 (0.75-1.00)	1.29 (1.06-1.58)	1.41 (1.19-1.66)

Educational level	No studies	1	1	1
	
	Primary education completed	1.15 (0.99-1.33)	1.64 (1.34-2.00)	1.28 (1.08-1.51)
	
	Secondary education or more	1.13 (0.90-1.42)	1.48 (1.05-2.08)	1.28 (0.97-1.68)

Self rated health	Very good/good	1	1	1
	
	Fair/poor/very poor	1.13 (0.97-1.32)	0.22 (0.16-0.29)	0.19 (0.16-0.24)

Nª of chronic conditions	None	1	1	1
	
	1	0.89 (0.74-1.08)	1.50 (1.11-2.04)	1.33 (1.04-1.71)
	
	≥2	0.74 (0.61-0.89)	1.19 (0.90-1.57)	0.93 (0.74-1.17)

Number of medications	None	1	1	1
	
	1	1.28 (0.96-1.72)	1.02 (0.56-1.84)	0.83 (0.52-1.30)
	
	≥ 2	1.15 (0.87-1.53)	0.58 (0.33-1.02)	0.48 (0.31-0.72)

BMI	Normal	1	1	1
	
	Overweight	0.98 (0.84-1.15)	0.99 (0.79-1.27)	0.97 (0.79-1.18)
	
	Obesity	0.77 (0.63-0.95)	0.60 (0.46-0.77)	0.56 (0.45-0.70)

Smoking habits	Smoker	1	1	1
	
	Ex Smoker	1.03 (0.60-1.77)	0.59 (0.24-1.45)	1.20 (0.60-2.42)
	
	Non Smoker	1.37 (0.90-20.7)	0.51 (0.25-1.04)	0.86 (0.50-1.48)

Sleep habits (hours/day)	< 8	1	1	1
	
	≥ 8	1.01 (0.88-1.15)	1.19 (0.99-1.43)	1.35 (1.15-1.58)

SNHS	1987	1	-	-
	
	1993	1.82 (1.40-2.36)	1	1
	
	1995-97	2.82 (2.15-3.70)	0.98 (0.65-1.49)	1.44 (1.02-2.05)
	
	2001	3.66 (2.86-4.67)	1.15 (0.78-1.69)	1.70 (1.24-2.34)
	
	2003	2.53 (1.99-3.21)	1.20 (0.84-1.72)	1.59 (1.19-2.13)
	
	2006	3.69 (2.89-4.70)	1.29 (0.90-1.86)	1.63 (1.21-2.19)

The results of the logistic models are shown as adjusted odds ratios (ORs) with 95% confidence intervals. Models adjusted by all variables shown in the table, no significant interactions were found

Among men, LTPA has also significantly increased from 1987 to 2006 (P < 0.001, Figure 1), but no significant changes for physical fitness were observed (Figures 2, 3). The results of the multivariate analysis to estimate time trends and associated factors for older men are found within table 5. Factors associated to less practicing LTPA in men were: age ≥ 80 years, being married, and obesity. Variables associated with worse physical fitness among men were the same as for women: age 80 years or over, worse self-rated health, ≥ 2 medications, and obesity (only for walking for one hour).

**Table 5:** Logistic Regression Models for MEN

		Leisure time physical activity	Walking up 10 step	Walking for an hour
Age group	65-79	1	1	1
	
	≥ 80	0.61 (0.49-0.75)	0.37 (0.28-0.49)	0.30 (0.24-0.39)

Marital status	Unmarried/widow/divorced	1	1	1
	
	Married or living with couple	0.79 (0.65-0.95)	0.74 (0.54-1.02)	0.94 (0.73-1.22)

Educational level	No studies	1	1	1
	
	Primary education completed	1.27 (1.04-1.54)	1.68 (1.25-2.26)	1.46 (1.13-1.88)
	
	Secondary education or more	1.13 (0.88-1.44)	1.64 (1.06-2.57)	1.29 (0.92-1.81)

Self rated health	Very good/good	1	1	1
	
	Fair/poor/very poor	0.91 (0.76-1.08)	0.17 (0.11-0.25)	0.16 (0.12-0.22)

Nª of chronic conditions	None	1	1	1
	
	1	1.10 (0.87-1.37)	1.45 (0.95-2.20)	1.52 (1.06-2.16)
	
	≥2	1.13 (0.88-1.44)	1.12 (0.75-1.66)	0.97 (0.68-1.38)

Number of medications	None	1	1	1
	
	1	1.00 (0.76-1.32)	1.25 (0.57-2.71)	0.63 (0.35-1.15)
	
	≥ 2	0.89 (0.66-1.18)	0.35 (0.18-0.71)	0.33 (0.19-0.59)

BMI	Normal	1	1	1
	
	Overweight	1.00 (0.84-1.22)	1.40 (1.02-1.91)	1.08 (0.83-1.41)
	
	Obesity	0.66 (0.43-0.96)	0.92 (0.62-1.36)	0.70 (0.51-0.98)

Smoking habits	Smoker	1	1	1
	
	Ex Smoker	0.90 (0.72-1.13)	0.54 (0.33-0.87)	0.95 (0.66-1.36)
	
	Non Smoker	0.79 (0.62-1.02)	0.66 (0.39-1.12)	1.41 (0.95-2.09)

Sleep habits (hours/day)	< 8	1	1	1
	
	≥ 8	0.99 (0.84-1.17)	1.24 (0.93-1.65)	1.27 (1.00-1.60)

SNHS	1987	1	-	-
	
	1993	2.51 (2.03-3.11)	1	1
	
	1995-97	3.35 (2.65-4.23)	1.01 (0.57-1.78)	0.81 (0.51-1.26)
	
	2001	3.76 (3.04-4.66)	1.35 (0.89-2.02)	0.96 (0.69-1.35)
	
	2003	1.99 (1.62-2.45)	1.18 (0.80-1.74)	1.01 (0.73-1.41)
	
	2006	4.22 (2.85-5.59)	1.09 (0.74-1.62)	0.81 (0.58-1.13)

The results of the logistic models are shown as adjusted odds ratios (ORs) with 95% confidence intervals. Models adjusted by all variables shown in the table, no significant interactions were found.

## Discussion

Our study revealed an increase in LTPA from 1987 to 2006 in older Spanish people. The results are consistent with studies conducted in European, American and Asian countries [22,25,27]. In Spain, the study conducted by Roman-Viñas et al [40] observed a slight decreased in the proportion of sedentary leisure time activities for males (from 50% to 45%) and females (from 67% to 63%). However, this study was conducted in Catalonia, a region of Spain, and did not focus in LTPA in older people [40,41]. Therefore, our study is the first one that includes national data over a period of 20 years in the Spanish older population.

The Scottish Health Survey found an increase in PA among older people aged between 65 to 74 years, but a decline in walking (65-74 year) and training sports among 75 years and over [22]. The results derived from the Health Survey in England (1991-2004) found an upward trend in regular sports participation in all age groups, but particularly pronounced among the older groups (≥ 65 years) [27]. The Behavioral Risk Factors Surveillance System found an increase in the prevalence of walking from 1987 to 2000, particularly in older people [28]. The Japan Collaborative Cohort Study also showed an increase in sports and physical exercise in subjects aged 50-79 years old [25]. Previous studies have shown a tendency that decreased activity occurs with increasing age [14,24,31]; however, a cross-sectional study conducted with Chinese women found that older age was positively associated with participation in exercise/sports and walking [17].

We have also found that women exhibit lower prevalence of LTPA and physical fitness as compared to men in all surveys, which is in agreement with the results by Stamatakis et al [27]. The Cardiovascular Health Study showed that men were more active in LTPA than women in all age groups [6], which also agree with the current results. Contrary, Simpson et al. [28] have shown a higher prevalence of elder women who walk. In this study, women were two to three times more likely than men to report that walking was one of their LTPA.

The decreased prevalence of LTPA among women can be attributed to monitorization of daily transports [26]. Gallant and Dorn [42] have reported that social network emerged more importantly for women than for men, which indicates that women may perform many of health behaviors within a social context [43]. The omission of household activities may underestimate the total PA within women and result in misclassified as physically not very active [26]. Further, cultural perspectives can influence LTPA [44]. In fact, Spanish people have a poor attitude to change or improve their physical activity as compared to Europeans [45]. In Spain, gender differences in LTPA are in accordance with findings previously reported by Cornelio et al [41].

We found that age ≥80 years, to be married, ≥ 2 co-morbid chronic conditions and obesity were associated with a lower likelihood of reporting LTPA in both genders, which is in agreement with previous studies conducted in Australia [16] and USA [29]. Gallant and Dorn [42] reported that marital status showed an influential element in men's health behavior. Our results are also consistent with Kaplan et al [29] who found that married subjects were less likely to be active than single, widowed, or divorced.

The current study also found that education level (primary or over) was related with LTPA and fitness activity, which agrees with previous studies [14,17,32]. The Shanghai Women's Health Study reported that women aged 40-70 years of age with more education were more likely to practice sports, but widows/divorced/separated were more likely to walk [17]. In contrast, Wong et al reported that people with lower educational level spent more time on walking than those with higher level [32].

Among behavioral factors, smoking and BMI > 28 were negatively associated with LTPA. These results agree with previous studies showing that obesity was associated with lower activity [22,46]. In the longitudinal analyses of the CHIANTI study, obese older population with low muscle strength had steeper decline in walking speed, walk 400 m or climb one flight on stairs as compared with those without obesity or low muscle strength [47]. Spanish sedentary older people exhibited lower education level in both genders. Sedentary men consume alcohol less frequently and have a higher number of chronic diseases than women, while sedentary older women are obese, have never smoked and consumed more frequently 3 or more drugs than men [34].

Our results provide evidence that older people reporting a fair/poor/very poor self-rated health status have difficulties in walking and climbing stairs. Self-perceived health status is considered as a reliable predictor of PA, walking decline and mortality in older people [6]. In fact, perceived poor health status has been associated with lower PA [33], as PA significantly correlates with self-reported health in older adults [48].

Our study has revealed an increase in LTPA during the last 20 years, but not for the capacity to walk up ten steps or walk for one hour. The tendency to respond affirmatively to LTPA can be explained because older people sometimes have a negative opinion of those inactive, and have their own beliefs about the effects of PA [49]. In addition, the less active older individual tends to underestimate the benefits of exercise [50]. This may be related to the fact that this group is determined by anti-aging messages that appear in mass media [51], social [42] and cultural contexts [44]. They may have a tendency to integrate socially, avoiding showing they need help for anything [52]. In addition, elders may overestimate the PA [18] which they practiced, or be unaware of the recommendations or levels of exercise for effective results [14]. Other factors that may influence adherence to PA in the elderly are outcome expectations and environmental barriers [53], self-efficacy [54]. It is also possible that older people meet the PA recommendations to maintain their health status but at the same time have a sedentary lifestyle, and therefore their physical fitness has not improved.

Finally, we should recognize some limitations of our study. First, discrepancies between trends of increase or decrease in PA among studies may be related to the definition and measurement of LTPA and physical fitness [18], study designs, or the statistical analysis [51]. In the current study, we used a self-reported measure of PA including two questions with 2 possible answers, which can have limited the assessment of activity and exercise. Additionally, the SNHS only assessed LTPA and PA; therefore, we cannot examine occupational, recreational, and transport-related PA independently. In addition, the validity of the questions included in the surveys have not been analyzed. The use of objective measures could complement self-report data to avoid bias, i.e., quantification of physical activity level by calculating MET or using accelerometers [27]; however, this is not generally feasible in large-scale population surveys due to extensive costs. Further, even when individuals can overestimate their participation in exercise, and underestimate sedentary behaviors [14,18], surveys are extremely useful for investigating patterns, frequencies, and time trends. Finally, the use of objective measurements for assessing PA has changed over the last years, so the use of the same outcome for 20 years is difficult. Secondly, the study design does not permit to establish a cause and effect relationship due to the lack of longitudinal follow-up of the same individuals. Nevertheless, the use of a national population-based survey permits the inclusion of representative national sample sizes. Despite these limitations this study provides additional insight into demographic aspects of LTPA and physical fitness in older adults for whom there is little information at population levels, particularly in Spain.

## Conclusion

Our study revealed an increase in LTPA, but not in physical fitness, from 1987 to 2006 in older Spanish people. Older people (age ≥ 80 years), married, with a greater number of co-morbid chronic conditions and obese exhibited a relatively lower LTPA. Similarly, older people (age ≥ 80 years, those taking a greater number of medications for chronic conditions, obese, and with worse self-perceived health status tended to have a relatively lower physical fitness. These results have potential implications for health services, as identification of these factors can help to prevent physical inactivity and improve the health status of older people in Spain

## List of abbreviations

PA: Physical activity; LTPA: Leisure time physical activity; SNHS: The Spanish National Health Surveys; ORs: Odds ratios.

## Competing interests

The authors declare that they have no financial competing interests and non-financial competing interests.

Conflict of interest**: **The manuscript, or parts of it, have not been and will not be submitted elsewhere for publication.

Role of the funding source: We have not financial interest and we have not received direct o indirect funding, and there is not conflict of interest.

## Authors' contributions

DPC conceived of the study, and participated in its design and coordination and draft the manuscript. CAB carried out the acquisition of the data, analysis and interpretation of data. She has been involved in revising it critically. VHB participated in the design of the study and performed the statistical analysis. PCG carried out the acquisition of the data, analysis and interpretation of data. She has been involved in revising it critically. RJG participated in the design of the study and performed the statistical analysis. EPM carried out the acquisition of the data, analysis and interpretation of data. She has been involved in revising it critically. CFP conceived of the study, and participated in its design and coordination and helped to draft the manuscript.

All authors read and approved the final manuscript.

## Authors' information

None

## Pre-publication history

The pre-publication history for this paper can be accessed here:

http://www.biomedcentral.com/1471-2458/11/799/prepub
